# Does Sarcopenia Accompanying End-Stage Knee Osteoarthritis Affect the Outcomes following Total Knee Arthroplasty?

**DOI:** 10.3390/medicina59061078

**Published:** 2023-06-02

**Authors:** Oog-Jin Shon, Gi Beom Kim, Seung Jae Cho

**Affiliations:** 1 Department of Orthopedic Surgery, Yeungnam University College of Medicine, 170 Hyonchung-ro, Namgu, Daegu 42415, Republic of Korea; maestro-jin@hanmail.net; 2 Department of Orthopedic Surgery, Yeungnam University Medical Center, 170 Hyonchung-ro, Namgu, Daegu 42415, Republic of Korea; kimjsj1021@naver.com

**Keywords:** end-stage osteoarthritis, total knee arthroplasty, sarcopenia, patient-reported outcome measures

## Abstract

*Background and Objectives:* This study aimed to investigate the prevalence of sarcopenia in patients undergoing total knee arthroplasty (TKA) for advanced knee osteoarthritis (OA), and to assess whether sarcopenia accompanying OA affects patient-reported outcome measures (PROMs) after TKA. We evaluated which predisposing factors could influence the development of sarcopenia in patients with advanced knee OA. *Material and Methods:* A total of 445 patients whose body composition, muscle strength, and physical performance could be measured before primary TKA were enrolled. Sarcopenia was defined according to the Asian Working Group for Sarcopenia 2019 criteria. Patients were categorized into sarcopenia (S, n = 42) and non-sarcopenia groups (NS, n = 403). PROMs were investigated using the Knee Injury and Osteoarthritis Outcome Score and Western Ontario and McMaster Universities Osteoarthritis Index. Additionally, postoperative complications and predisposing factors for sarcopenia were evaluated. *Results:* The incidence of sarcopenia in the entire sample was 9.4%; the prevalence was higher in men (15.4%) than in women (8.7%), and significantly increased with advancing age (*p* < 0.001). At the six–month follow-up, PROMs in group S were significantly inferior to those in group NS, except for the pain score; however, at the 12-month follow-up, no significant difference was observed between the groups. Multivariate logistic regression indicated that age, body mass index (BMI), and a higher modified Charlson Comorbidity Index (mCCI) were predisposing factors for sarcopenia. *Conclusions:* A higher prevalence of sarcopenia was observed in men with progressive knee OA. Up to six months after primary TKA, PROMs in group S were inferior to those in group NS, except for the pain score; however, no significant difference was observed between the groups at 12 months. Age, BMI, and higher mCCI were predisposing factors for sarcopenia in patients with OA.

## 1. Introduction

Osteoarthritis (OA) is a destructive joint disease that cause cartilage degeneration, osteophyte formation, and changes in the periarticular bones, resulting in disability [1]. Total knee arthroplasty (TKA) is an effective treatment for end-stage OA of the knee joint [2,3], and its use has increased globally over the past few decades [4,5,6,7].

The role of muscles in maintaining functional performance to prevent falls and related fragility fractures has been emphasized [8,9]. Skeletal muscles naturally degenerate with age, and this loss accelerates after the age of 65 years, with a risk of adverse outcomes such as physical disability, poor quality of life, and death [10,11]. This condition is called sarcopenia, and is characterized by decreased muscle mass and strength and impaired muscle function [12].

Sarcopenia and OA, which are clinically important and common pathological states, have become increasingly significant with the increase in the population of older adults [13,14]. In skeletal tissues, muscles and joints interact both mechanically and functionally. Therefore, aging and various pathological states simultaneously influence the muscles and joints [15,16]. Sarcopenia is an indicator of frailty and loss of independence in elderly individuals. Furthermore, it is associated with increased physical disability, resulting in an increased risk of falls. Patients with end-stage OA necessitating TKA have significant impairments in walking and daily living due to pain. Therefore, weakness in the muscles of the lower extremities, including the quadriceps, is inevitable [17,18]. Furthermore, sarcopenia caused by OA may affect the outcomes after TKA. However, there is a paucity of literature on the impact of sarcopenia on the outcomes of TKA.

This study aimed to investigate the prevalence of sarcopenia in patients undergoing TKA for end-stage knee OA, and whether sarcopenia accompanying OA affects patient-reported outcome measures (PROMs) after TKA. We attempted to answer the following questions:

1. What is the incidence of sarcopenia in patients undergoing TKA for advanced knee OA?
2. Are the PROMs in patients with sarcopenia associated with knee OA inferior to those in patients without sarcopenia?
3. If yes, what are the predisposing factors for sarcopenia in patients with advanced knee OA?

## 2. Materials and Methods

### 2.1. Participants

**Figure 1:**
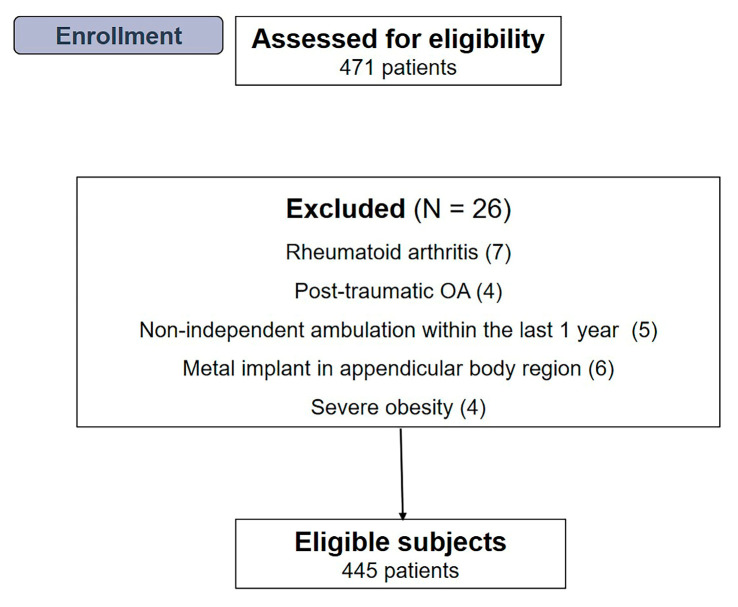
Flow diagram illustrating patient enrollment. Overall, 445 patients were enrolled in our study.

The medical records of 471 consecutive patients (665 knees) who underwent primary TKA between May 2020 and August 2021 were screened. Inclusion criteria for this study were as follows: (1) patients with symptomatic advanced OA (Kellgren—Lawrence [K—L] grade ≥ 3), (2) age > 60 years, (3) having a minimum follow-up period of 12 months, and (4) whose body composition, muscle strength, and physical performance could be measured before the index operation. All procedures were performed by two experienced surgeons using the same cemented and posterior-stabilized implants. Patients with other types of diagnosis (rheumatoid arthritis, n = 7; post-traumatic OA, n = 4), non-independent ambulation within the previous one year due to other comorbid medical conditions (n = 5), metal implants in the appendicular body parts that could potentially affect the accuracy of appendicular skeletal muscle mass measurement (n = 6), or severe obesity (body mass index [BMI] > 35 kg/m^2^; n = 4) were excluded. Finally, 445 patients, including 190 who underwent bilateral primary TKA, were included in the analysis (Figure 1). This retrospective study was approved by the institutional review board of our hospital prior to gathering patient data.

### 2.2. Anthropometric Measurements

Body composition was measured using whole-body dual-energy X-ray absorptiometry (DXA) (Horizon, Hologic, Bedford, MA, USA) to calculate the skeletal muscle mass index (SMI). As absolute muscle mass correlates with height, SMI was calculated by correcting muscle mass with height (lean mass/height^2^ [kg/m^2^]). The total appendicular SMI (ASMI) was calculated as the sum of the arm SMI (arm lean mass/height^2^ [kg/m^2^]) and leg SMI (leg lean mass/height^2^ [kg/m^2^]) [19].

### 2.3. Definition of Sarcopenia

**Figure 2:**
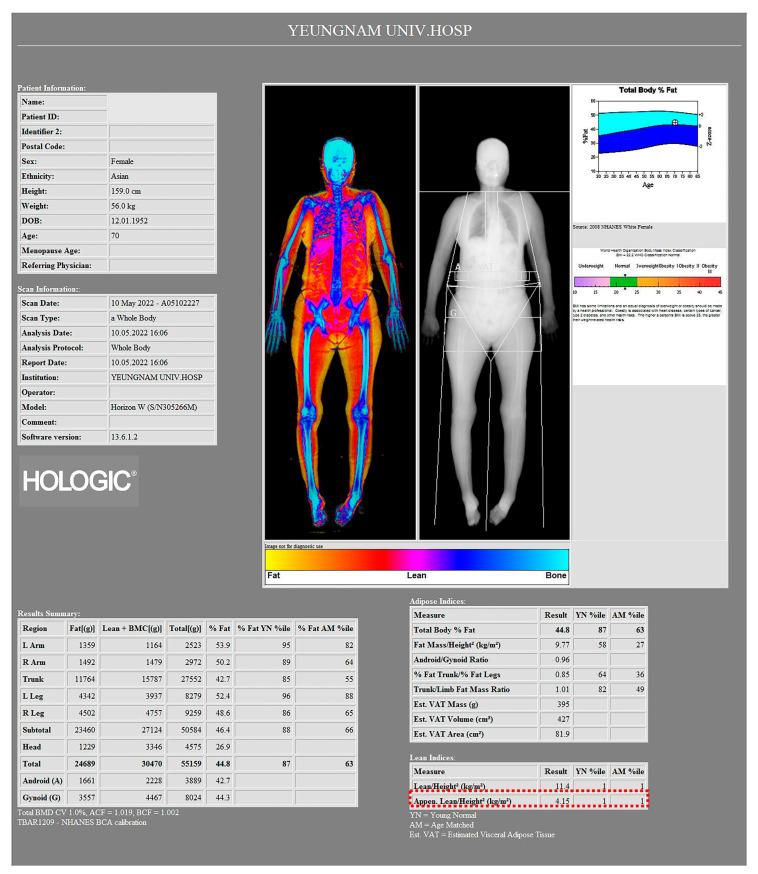
Image of a 70-year-old female patient from a body composition measurement made by a whole-body dual-energy X-ray absorptiometry (DXA) scanner. According to the Asian Working Group for Sarcopenia (AWGS) 2019 diagnostic criteria, ASMI of this patient was 4.15 kg/m^2^ (red dotted box), which can be considered as a low muscle mass.

Sarcopenia was defined based on the Asian Working Group for Sarcopenia (AWGS) 2019 diagnostic criteria [20]. Individuals with low muscle mass, muscle strength, and/or physical performance were classified as sarcopenia. Low muscle mass was defined as an ASMI < 5.4 kg/m^2^ for women and <7.0 kg/m^2^ for men [20] (Figure 2).

Muscle strength was assessed with a handgrip strength test using a handgrip dynamometer (Jamar, Bolingbrook, IL, USA) by applying the Southampton protocol [21]. Low muscle strength was defined as a handgrip strength <28 kg for men and <18 kg for women [20]. Physical performance was assessed using the six-meter walking speed test. Low physical performance was defined as a gait speed <1.0 m/s for both men and women [20]. Based on these criteria, the incidence of sarcopenia in patients with advanced OA was determined, and patients were classified to the sarcopenia group (group S) or the non-sarcopenia group (group NS).

### 2.4. Outcome Assessments

Demographic characteristics (including age, sex, BMI, follow-up period, and modified Charlson Comorbidity Index [mCCI] [22]) and laboratory data (including hemoglobin [Hb, g/dL] and total protein [g/dL] levels) were investigated before the index surgery. mCCI is a valuable preoperative risk assessment tool for patients undergoing surgery [22]. It was calculated by summing the weighted scores for the associated comorbidities (Table 1).

The prevalence of sarcopenia according to age was evaluated to identify age-related characteristics.

PROMs were assessed using the Knee Injury and Osteoarthritis Outcome Score (KOOS) [23] and the Western Ontario and McMaster Universities Osteoarthritis Index (WOMAC) [24]. The range of motion (ROM) of the knee joint (including flexion contracture and further flexion) was measured using a standardized manual goniometer with a 30 cm plastic movable long arm [25]. Patients were regularly followed up postoperatively at six weeks and three, six, and 12 months. All clinical evaluations were compared between the two groups.

Further, the incidence of postoperative complications was compared between the groups. In addition to surgery-related complications such as postoperative blood transfusion or periprosthetic joint infection (PJI), systemic complications were investigated. Blood transfusion was performed in patients whose Hb levels dropped to <7.0 g/dL within 2 weeks of the index surgery [26]. PJI was diagnosed according to the latest evidence-based criteria from the International Consensus Meeting [27]. Systemic complications were defined as the exacerbation of underlying systemic comorbidities or the development of new medical problems [28].

To identify the predisposing factors for sarcopenia in patients with end-stage knee OA, all variables were assessed using univariate and multivariate logistic regression analyses.

### 2.5. Postoperative Protocols

All patients underwent the same rehabilitation protocol. The drain was removed 24 h after surgery. A perioperative pain-control protocol was performed, including a multimodal drug regimen, postoperative patient-controlled analgesia, and intraoperative periarticular injections. Active and passive postoperative ROM exercises were initiated on the day of surgery. If the acute pain was tolerable, partial weightbearing with a crutch was allowed on the first postoperative day. Full weight-bearing was permitted 3 weeks after surgery.

### 2.6. Statistical Analysis

Statistical evaluation was performed using SPSS software version 28 (IBM Corp, Armonk, NY, USA), and continuous data are expressed as means ± standard deviation with ranges. All dependent variables were tested for normality of distribution and equality of variance using the Kolmogorov–Smirnov test. A two-sided Pearson’s χ2 test or Fisher’s exact test were used to compare the ratios between the groups. An independent samples *t*-test was used to determine significant differences between the two groups. Univariate and binary logistic regression analysis were performed on categorical and continuous variables to determine the predisposing factors for sarcopenia in patients with end-stage knee OA. All variables, including demographic data, preoperative laboratory data, and clinical outcomes, were analyzed individually using the univariate regression analysis. A multivariate binary logistic regression analysis was subsequently performed to identify the predisposing factors for sarcopenia while accounting for potential confounding variables. Odds ratios (ORs) and 95% confidence intervals (CIs) were calculated using binary logistic regression analyses. Statistical significance was set at *p* < 0.05.

## 3. Results

A total of 445 patients (393 women, 52 men) were included in this study. The average age at operation was 71.8 years (range, 60–88 years), and the average follow-up period was 14.5 months (range, 12.0–26.0 months). According to the AWGS 2019 diagnostic criteria, the overall prevalence of sarcopenia in this cohort was 9.4% (34/363), and the prevalence was higher in men (8/52, 15.4%) than in women (34/393, 8.7%). Baseline characteristics and sarcopenia parameters are summarized in Table 2.

The prevalence of sarcopenia increased significantly with age (*p* < 0.001) (Table 3).

At the six-month follow-up, group S showed significantly worse PROMs than group NS, except for the pain score; however, at the 12-month follow-up, there was no significant difference between the groups (Table 4).

More patients in group S received postoperative blood transfusions (*p* < 0.001). PJI occurred significantly more frequently in group S (acute postoperative infection, one; acute hematogenous infection, one) (*p* = 0.009). There was no significant difference in systemic complications between the two groups (Table 5).

The results of the logistic regression analysis are presented in Table 6. The univariate binary logistic regression showed that age (OR, 1.5; 95% CI, 0.8–1.7; *p* < 0.001), BMI (OR, 0.8; 95% CI, 0.6–0.9; *p* < 0.001), preoperative levels of Hb (OR, 0.8; 95% CI, 0.6–0.9; *p* = 0.021) and total protein (OR, 0.7; 95% CI, 0.5–0.8; *p* = 0.037), and higher mCCI (OR, 1.1; 95% CI, 0.8–1.4; *p* < 0.001) were associated with sarcopenia. The multivariate binary logistic regression analysis to determine each variable’s independent contribution to the sarcopenic status after accounting for confounding factors showed that age (OR, 1.4; 95% CI, 0.9–1.8; *p* < 0.001), BMI (OR, 0.7; 95% CI, 0.6–0.9; *p* = 0.019), and higher mCCI (OR, 1.2; 95% CI, 0.8–1.5; *p* = 0.039) were risk factors for sarcopenia.

## 4. Discussion

The most notable findings of the present study were that a higher prevalence of sarcopenia was observed in men with progressive knee OA. Although PROMs in group S were inferior to those in group NS up to six months after primary TKA, no significant difference was observed between the groups postoperatively at 12 months. In addition, age, BMI, and higher mCCI were predisposing factors for sarcopenia in patients with progressive knee OA.

To date, few studies have reported the prevalence of sarcopenia in patients with knee OA [29,30]. However, there is a paucity of literature regarding the prevalence of sarcopenia in patients undergoing TKA for advanced OA. According to the diagnostic criteria and age, the incidence of sarcopenia in community-dwelling elderly individuals has been reported to vary from 5 to 50% [31] and 0.1 to 23.6% [17]. The most important finding of the present study was that the overall prevalence of sarcopenia was 9.4%, which did not differ significantly from the prevalence in the general community-dwelling elderly population. Therefore, considering that sarcopenia often occurs in patients undergoing TKA for advanced arthritis, clinical outcomes can be predicted by diagnosing sarcopenia through preoperative anthropometric measurements and handgrip strength. Furthermore, interventions to improve sarcopenia as a modifiable factor should be considered.

In the present study, group S showed significantly inferior PROMs up to six months after TKA, and there was no significant difference between the two groups at 12 months postoperatively. This indicates that sarcopenic OA may have adversely affected early clinical outcomes after TKA. This is the first study to report that sarcopenia affects the outcome of TKA. Although a recent study reported the prevalence of sarcopenia and its effect on clinical outcomes in patients who underwent TKA for end-stage OA [17], it only suggested that TKA could lead to significant improvements in clinical outcomes, even in patients with sarcopenic OA and did not compare clinical outcomes after TKA according to the presence of sarcopenia. Moreover, despite similar demographic characteristics compared to the present study, the reported incidence of sarcopenia in that study was 32.8% (19/58), which may be attributed to the relatively small sample size.

In terms of postoperative complications, patients in group S received postoperative blood transfusion more frequently. This is consistent with the results of a recent study that diagnosed sarcopenia based on bioelectrical impedance analysis and reported that patients with sarcopenia who underwent TKA for sarcopenic OA were more likely to require postoperative blood transfusion. This may be due to the lower preoperative Hb and total protein levels in group S in that study.

In addition, PJI was reported in two patients in group S. In a study that diagnosed sarcopenia using the psoas-lumber vertebral index, sarcopenia was reported to be a risk factor for PJI after TKA [32]. Further studies on sarcopenia as an optimizable predisposing factor for PJI after TKA are required.

Previous studies have reported that age, low BMI, and lower activity levels influence the progression of sarcopenia-related OA [29,30,33]. Severe knee pain caused by advanced OA may impair physical function and increase the risk of sarcopenia. The increase in the prevalence of sarcopenia with age in the present study supports this finding. In addition, the prevalence of sarcopenia was higher in men than in women (15.4% vs. 8.7%), which may be attributed to a significant decrease in physical activity owing due to advanced knee OA in men, who were usually more active than women.

A lower BMI is a risk factor for sarcopenia [33,34]. As ASMI is positively correlated with BMI and it is well known that obesity or sarcopenic obesity increases the risk of arthritis, the relationship between BMI and sarcopenia is contentious [35]. In the present study, lower BMI was associated with an increased risk of sarcopenia. However, BMI is a modifiable factor that can be improved through dietary interventions. Therefore, further studies on improving the BMI in patients with advanced OA-related sarcopenia are required.

Despite these informative results, this study had some limitations. First, this was a retrospective study with a short-term follow-up. Although the recovery of muscle mass or muscle strength after TKA could not been confirmed, clinical outcomes continuously improved after TKA and were maintained for 12 months after surgery. A follow-up period of approximately 12 months can be considered reasonable for the recovery of muscle mass or strength. Second, it was not possible to evaluate the degree of objective recovery after surgery because additional DXA and handgrip strength evaluations were not performed for muscle mass and muscle strength after TKA, respectively. Although there may be additional medical costs or insurance issues, future research should objectively evaluate data on recovery using additional anthropometric measurements. Third, the cohort in the present study was not representative of all grades of knee OA. Sarcopenic parameters and clinical outcomes may vary in patients with mild arthritis. This study provides meaningful information on the effects of muscle mass or strength on the outcomes in patients who underwent TKA for advanced knee OA. Finally, women were predominant in the present study. Because the prevalence of knee OA in women is much higher in Asia, women usually undergo TKAs more frequently than men [36]. Moreover, since the prevalence of sarcopenia and knee OA differed by gender, several factors leading to the progression of OA independently of sarcopenia may need to be considered. Particularly, the relationship between obesity and sarcopenia is controversial, and the variables may have different effects depending on ethnicity [29,35]. Therefore, the results of this study may not be generalizable to other ethnic groups.

Nevertheless, our study had important strengths. This study assessed the incidence of sarcopenia in patients with progressive knee OA and presented the differences according to sex and age group. Furthermore, the predisposing factors for sarcopenia in these patients were investigated.

## 5. Conclusions

A higher prevalence of sarcopenia was observed in men with progressive knee OA. Up to six months after primary TKA, PROMs in group S were inferior to those in group NS, except for the pain score; however, no significant difference was observed between the groups at 12 months. Age, BMI, and higher mCCI were predisposing factors for sarcopenia in patients with OA.

## Figures and Tables

**Table 1:** Scoring system for the modified Charlson Comorbidity Index (mCCI).

Variables	Total
Peripheral vascular disease or pain at rest	1
Congestive heart failure	1
Prior MI	1
DM	1
Prior transient ischemic attack or stroke	1
COPD	1
Renal failure	2
Hemiplegia	2
Ascites or esophageal varices	3
Disseminated cancer	6
≤40 yrs old	0
41–51 years old	1
51–60 years old	2
61–70 years old	3
≥70 years old	4

NOTE. MI, myocardial infarction; DM, Diabetes mellitus; COPD, chronic obstructive pulmonary disease. The mCCI was calculated by summing the weighted scores for the associated comorbidities.

**Table 2:** Baseline characteristics according to the presence or absence of sarcopenia.

Variables	Total N = 445 (100%)	Group S N = 42 (9.4%)	Group NS N = 403 (90.6%)	*p* Value
Age, years ^1^	71.8 (60–88)	76.2 (69–88)	67.8 (60–76)	**<0.001 ** ^4^
Sex, n ^2^ Female, n Male, n				
	393 (88.3)	34 (83.3)	359 (88.8)	0.129 ^5^
	52 (11.7)	8 (16.7)	44 (11.2)	
BMI, kg/m^2 3^	26.9 ± 3.5	23.2 ± 3.2	27.6 ± 3.1	**<0.001 ** ^4^
F/U period, months ^1^	14.5 (12–27)	12.4 (12–27)	12.3 (12–27)	0.871 ^4^
Bilaterality, n ^2^	190 (42.7)	17 (40.5)	173 (42.9)	0.870 ^5^
Preop K-L grade, n ^2^ Grade III, n Grade IV, n				
	112 (25.2)	11 (26.2)	101 (25.1)	0.853 ^5^
	333 (74.8)	31 (73.8)	302 (74.9)	
mCCI, n ^2^				
2	18 (4.0)	-	18 (4.5)	
3 and 4	210 (47.2)	3 (7.1)	207 (51.4)	
5–8	156 (35.1)	20 (47.6)	136 (33.7)	
≥9	61 (13.7)	19 (45.2)	42 (10.4)	**<0.001 ** ^5^
Hb level, (g/dL) ^3^	12.6 ± 1.9	11.8 ± 2.5	12.9 ± 1.6	**0.021 ** ^4^
Total protein, (g/dL) ^3^	6.7 ± 1.2	6.1 ± 1.6	6.9 ± 1.0	**0.037 ** ^4^
ASMI (ASM/height^2^), kg/m^2 3^	5.7 ± 0.6	5.2 ± 0.4	6.1 ± 0.6	**<0.001 ** ^4^
Grip Strength, kg ^3^	17.9 ± 3.2	16.2 ± 3.2	19.0 ± 3.2	**<0.001 ** ^4^
6 m walking speed, m/s ^3^	1.1 ± 0.2	0.8 ± 0.2	1.3 ± 0.2	**0.018 ** ^4^

NOTE. ^1^ Data are presented as number with range. ^2^ Data are presented as number with percentage. ^3^ Data are presented as means ± standard deviations. ^4^ Independent samples *t*-test was used to compare the difference in clinical outcomes between the groups. ^5^ Fisher’s exact test was used to compare the ratios between the groups. The level of statistical significance was set at *p* < 0.05. BMI, body mass index; f/u, follow-up; mCCI, modified Charlson Comorbidity Index; Hb, hemoglobin; ASMI, appendicular skeletal muscle mass index.

**Table 3:** Prevalence of sarcopenia according to age group ^1^.

	Total	60–69 Years	70–79 Years	≥80 Years	*p* Value
Female	34/393 (8.7)	7/154 (4.5)	16/195 (8.2)	11/44 (25.0)	
Male	8/52 (15.4)	0/11	2/14 (14.3)	6/27 (22.2)	
Total	42/445 (9.4)	7/165 (5.5)	19/209 (9.1)	16/71 (22.5)	<0.001 ^2^

NOTE. ^1^ Data are presented as number with percentage. ^2^ Fisher’s exact test was used to compare the ratios between the groups. The level of statistical significance was set at *p* < 0.05.

**Table 4:** Comparison of clinical outcomes between the groups. (**A**) KOOS ^1^

Variables	Group S N = 42	Group NS N = 403	*p* Value ^2^
**KOOS Pain**			
Preop	43.8 ± 8.1	44.3 ± 8.5	0.738
PO at 3 months	80.7 ± 6.0	81.0 ± 7.9	0.681
PO at 6 months	82.1 ± 6.1	82.0 ± 6.5	0.731
PO at 12 months	84.1 ± 4.9	84.2 ± 5.1	0.542
**KOOS Symptom**			
Preop	47.2 ± 9.2	46.8 ± 8.1	0.814
PO at 3 months	60.1 ± 6.2	65.4 ± 6.1	**0.047**
PO at 6 months	69.4 ± 4.2	76.1 ± 4.9	**0.026**
PO at 12 months	71.4 ± 2.1	78.1 ± 3.9	0.061
**KOOS ADL**			
Preop	42.1 ± 6.8	42.8 ± 5.3	0.713
PO at 3 months	65.1 ± 8.2	75.4 ± 6.1	**<0.001**
PO at 6 months	73.8 ± 2.2	80.1 ± 2.7	**<0.001**
PO at 12 months	81.8 ± 2.2	83.1 ± 1.7	0.106
**KOOS Sport/Rec**			
Preop	33.2 ± 5.8	32.9 ± 3.9	0.523
PO at 3 months	45.1 ± 8.2	55.4 ± 5.1	**<0.001**
PO at 6 months	67.8 ± 3.2	77.1 ± 4.7	**<0.001**
PO at 12 months	75.1 ± 3.7	77.7 ± 4.2	0.720
**KOOS QOL**			
Preop	40.1 ± 7.1	40.7 ± 4.8	0.832
PO at 3 months	72.1 ± 9.1	78.7 ± 8.8	**<0.001 ^1^**
PO at 6 months	78.9 ± 5.0	84.1 ± 3.2	**<0.001 ^1^**
PO at 12 months	82.9 ± 1.9	82.1 ± 2.3	0.638
NOTE. ^1^ Data are presented as number ± standard deviation. ^2^ Independent samples *t*-test was used to compare the difference in clinical outcomes between the groups. The level of statistical significance was set at *p* < 0.05. KOOS, Knee Injury and Osteoarthritis Outcome Score; Preop, preoperatively; PO, postoperatively; ADL, activities of daily living; Rec, recreation; QOL, quality of life.			

(**B**) WOMAC ^1^			
**Variables**	**Group S** **N = 42**	**Group NS** **N = 403**	***p* Value ^2^**
**WOMAC Pain**			
Preop	9.6 ± 7.2	9.7 ± 4.1	0.614
PO at 3 months	6.6 ± 5.2	4.7 ± 4.1	**0.031**
PO at 6 months	3.7 ± 4.3	2.8 ± 2.9	**0.017**
PO at 12 months	2.4 ± 2.3	2.3 ± 2.9	0.532
**WOMAC Stiffness**			
Preop	5.2 ± 2.1	5.6 ± 2.7	0.729
PO at 3 months	3.8 ± 6.1	2.9 ± 4.7	**0.032**
PO at 6 months	3.3 ± 1.6	2.1 ± 1.9	**0.017**
PO at 12 months	2.3 ± 1.6	2.2 ± 1.9	0.817
**WOMAC Function**			
Preop	34.1 ± 5.5	33.8 ± 6.1	0.738
PO at 3 months	24.1 ± 9.5	15.1 ± 5.1	**<0.001**
PO at 6 months	19.2 ± 6.9	8.7 ± 2.3	**<0.001**
PO at 12 months	9.2 ± 2.9	7.7 ± 2.3	0.068
**WOMAC Total**			
Preop	48.9 ± 7.1	49.1 ± 4.6	0.828
PO at 3 months	34.5 ± 6.8	22.7 ± 4.9	**<0.001**
PO at 6 months	26.2 ± 5.8	13.6 ± 2.4	**<0.001**
PO at 12 months	11.2 ± 2.5	12.2 ± 2.3	0.272
NOTE. ^1^ Data are presented as number ± standard deviation. ^2^ Independent samples *t*-test was used to compare the difference in clinical outcomes between the groups. The level of statistical significance was set at *p* < 0.05. WOMAC, Western Ontario and McMaster Universities Osteoarthritis Index; Preop, preoperatively; PO, postoperatively.			

(**C**) ROM of the knee joint ^1^			
**Variables**	**Group S** **N = 42**	**Group NS** **N = 403**	***p* Value ** ^2^
**FC (°)**			
Preop	7.8 ± 2.9	8.3 ± 3.3	0.672
PO at 3 months	1.6 ± 4.2	1.7 ± 4.3	0.788
PO at 6 months	1.8 ± 1.9	1.9 ± 2.1	0.539
PO at 12 months	1.0 ± 0.9	0.8 ± 1.0	0.491
**FF (°)**			
Preop	117.9 ± 4.4	118.2 ± 4.6	0.821
PO at 3 months	129.8 ± 2.6	128.1 ± 3.1	0.423
PO at 6 months	135.3 ± 2.8	136.4 ± 2.9	0.625
PO at 12 months	139.8 ± 2.6	137.1 ± 2.9	0.717

NOTE. ^1^ Data are presented as number ± standard deviation. ^2^ Independent samples *t*-test was used to compare the difference in clinical outcomes between the groups. The level of statistical significance was set at *p* < 0.05. ROM, range of motion; FC, flexion contracture; Preop, preoperatively; PO, postoperatively; FF, further flexion.

**Table 5:** The incidence of systemic and specific complications ^1^.

		Total	Group S N = 42	Group NS N = 403	*p* Value ^2^
***Systemic***					
	Cardiovascular	8 (1.8)	1 (2.4)	7 (1.7)	0.551
	Pulmonary	6 (1.3)	1 (2.4)	5 (1.2)	0.450
	Gastrointestinal	-	-	-	-
	Hepatic	44 (9.8)	4 (9.5)	40 (9.9)	0.934
	Nephrotic	-	-	-	-
	Endocrinologic	-	-	-	-
	Urologic	87 (19.5)	8 (19.0)	79 (19.6)	0.931
	Cerebral	7 (1.6)	1 (2.4)	6 (1.5)	0.503
	Delirium	46 (10.3)	7 (16.7)	39 (9.7)	0.179
***Specific***					
	Blood transfusion	31 (7.0)	12 (28.6)	19 (4.7)	**<0.001**
	Venous thromboembolism				
	PTE	-	-	-	-
	DVT (proximal)	7 (1.6)	-	7 (1.7)	0.389
	DVT (distal)	42 (9.4)	4 (9.5)	38 (9.4)	0.984
	Infection	2 (0.4)	2 (4.8)	-	**0.009**
	Periprosthetic fracture	-	-	-	-

NOTE. ^1^ Data are presented as number (percentage). ^2^ Fisher’s exact test was used to compare the ratios between the groups. The level of statistical significance was set at *p* < 0.05. DVT was classified as proximal or distal DVT. Thrombi confined to the popliteal vein or above were classified as proximal DVT, and thrombi within calf vein were classified as proximal DVT. PTE, pulmonary thromboembolism; DVT, deep vein thrombosis.

**Table 6:** Univariate and multivariate logistic regression analyses for the presence of sarcopenia ^1^.

Variables	*p* Value		Odds Ratio (95% CI)	
	Univariate Analysis	Multivariate Analysis	Univariate Analysis	Multivariate Analysis
Sex	0.197		1.0 (0.3–1.6)	
Age	**<0.001** ^1^	**0.003** ^2^	1.5 (0.8–1.7)	1.4 (0.9–1.8)
BMI	**<0.001** ^1^	**0.019** ^2^	0.8 (0.6–0.9)	0.7 (0.6–0.9)
F/U period	0.965		1.0 (0.9–1.1)	
Bilaterality	0.892		1.2 (0.8–1.6)	
(Preop)				
K-L grade	0.914		1.2 (0.8–1.6)	
mCCI	**<0.001** ^1^	**0.039** ^2^	1.1 (0.8–1.4)	1.2 (0.8–1.5)
Hb level	**0.021** ^1^	0.371	0.8 (0.6–0.9)	0.7 (0.6–0.9)
Total protein	**0.037** ^1^	0.247	0.7 (0.5–0.8)	0.6 (0.5–0.8)
KOOS	0.126		1.0 (1.0–1.1)	
WOMAC	0.181		1.0 (0.9–1.0)	
ROM	0.802		1.2 (0.8–1.6)	

NOTE. ^1^ Univariate binary logistic regression analysis revealed that age, BMI, preoperative levels of Hb and total protein, and higher mCCI were associated with the presence of sarcopenia. ^2^ Multivariate binary logistic regression analysis showed that age, BMI, and higher mCCI were found to be predisposing risk factors for the presence of sarcopenia. BMI, body mass index; F/U, follow-up; K-L grade, Kellgren-Lawrence grade; mCCI, modified Charlson Comorbidity Index; Hb, hemoglobin; KOOS, the Knee Injury and Osteoarthritis Outcome Score; WOMAC, Western Ontario and McMaster Universities Osteoarthritis Index; ROM, range of motion.

## Data Availability

Data supporting the reported findings are available from the corresponding author upon reasonable request.
